# Brain metabolic differences between pure bulbar and pure spinal ALS: a 2-[^18^F]FDG-PET study

**DOI:** 10.1007/s00415-022-11445-9

**Published:** 2022-11-02

**Authors:** Antonio Canosa, Alessio Martino, Alessandro Giuliani, Cristina Moglia, Rosario Vasta, Maurizio Grassano, Francesca Palumbo, Sara Cabras, Francesca Di Pede, Filippo De Mattei, Enrico Matteoni, Giulia Polverari, Umberto Manera, Andrea Calvo, Marco Pagani, Adriano Chiò

**Affiliations:** 1 ALS Centre, ‘Rita Levi Montalcini’ Department of Neuroscience, University of Turin, Via Cherasco 15, 10126 Turin, Italy; 2 Azienda Ospedaliero-Universitaria Città Della Salute E Della Scienza Di Torino, SC Neurologia 1U, Turin, Italy; 3 Institute of Cognitive Sciences and Technologies, C.N.R., Rome, Italy; 4 Department of Business and Management, LUISS University, Rome, Italy; 5 Environment and Health Department, Istituto Superiore Di Sanità, Rome, Italy; 6 Positron Emission Tomography Centre AFFIDEA-IRMET S.P.A., Turin, Italy; 7 Neuroscience Institute of Turin (NIT), Turin, Italy; 8 Department of Medical Radiation Physics and Nuclear Medicine, Karolinska University Hospital, Stockholm, Sweden

**Keywords:** Amyotrophic lateral sclerosis, Positron emission tomography

## Abstract

**Background:**

MRI studies reported that ALS patients with bulbar and spinal onset showed focal cortical changes in corresponding regions of the motor homunculus. We evaluated the capability of brain 2-[^18^F]FDG-PET to disclose the metabolic features characterizing patients with pure bulbar or spinal motor impairment.

**Methods:**

We classified as pure bulbar (PB) patients with bulbar onset and a normal score in the spinal items of the ALSFRS-R, and as pure spinal (PS) patients with spinal onset and a normal score in the bulbar items at the time of PET. Forty healthy controls (HC) were enrolled. We compared PB and PS, and each patient group with HC. Metabolic clusters showing a statistically significant difference between PB and PS were tested to evaluate their accuracy in discriminating the two groups. We performed a leave-one-out cross-validation (LOOCV) over the entire dataset. Four classifiers were considered: support vector machines (SVM), K-nearest neighbours, linear classifier, and decision tree. Then, we used a separate test set, including 10% of patients, with the remaining 90% composing the training set.

**Results:**

We included 63 PB, 271 PS, and 40 HC. PB showed a relative hypometabolism compared to PS in bilateral precentral gyrus in the regions of the motor cortex involved in the control of bulbar function. SVM showed the best performance, resulting in the lowest error rate in both LOOCV (4.19%) and test set (9.09 ± 2.02%).

**Conclusions:**

Our data support the concept of the focality of ALS onset and the use of 2-[^18^F]FDG-PET as a biomarker for precision medicine-oriented clinical trials.

**Supplementary Information:**

The online version contains supplementary material available at 10.1007/s00415-022-11445-9.

## Introduction

Amyotrophic lateral sclerosis (ALS) is a fatal neurodegenerative disorder affecting motor neurons. In approximately two thirds of patients, the onset of symptoms occurs in spinal regions, mainly consisting of limb weakness. One third of cases show bulbar onset, characterized by impairment of speech and swallowing [1]. The spreading of motor impairment across body regions over time remains largely unpredictable at a single subject level. Ravits and La Spada proposed that neurodegeneration in ALS is a focal process which spreads contiguously along the three-dimensional anatomy of upper and lower motor neurons [2]. A number of magnetic resonance imaging (MRI) studies reported that patients with bulbar and spinal onset showed focal cortical changes in the corresponding regions of the motor homunculus [3–5]. 2-[^18^F]FDG-PET has been employed to investigate the brain metabolic changes associated with bulbar and spinal onset ALS, with inconsistent findings [6–8]. Indeed, regional hypo- and hypermetabolism demonstrated a very low accuracy in distinguishing spinal and bulbar onset patients [9]. Actually, neuroimaging studies focused on brain cortical changes in patients with pure bulbar and pure spinal impairment are lacking. Therefore, we aimed at evaluating the capability of brain 2-[^18^F]FDG-PET to disclose the cerebral metabolic features characterizing patients with exclusive bulbar or spinal motor impairment.

## Methods

### Participants

We screened for eligibility all consecutive patients diagnosed with definite, probable, and probable laboratory-supported ALS according to El Escorial revised diagnostic criteria [10] between 2009 and 2019 at the ALS Expert Centre of Turin, Italy, who underwent brain 2-[^18^F]FDG-PET at the time of diagnosis. The following demographic and clinical characteristics were collected: age at PET, sex, site of onset (spinal/bulbar), presence of *C9ORF72* hexanucleotide expansion, ALS Functional Rating Scale—Revised (ALSFRS-R), and King’s stage at PET. King’s stage was calculated from the ALSFRS-R score according to a published algorithm [11], and King’s stages 4a and 4b were combined as stage 4.

We classified as pure bulbar (PB) patients with bulbar onset who showed a score < 12 for the first three items of the ALSFRS-R at the time of PET at diagnosis (i.e. speech, salivation, and swallowing), and a normal score in the other items examining spinal functions (i.e. 36/36). Conversely, we considered as pure spinal (PS) patients with spinal onset, displaying a normal score (i.e. 12/12) in the bulbar items and a score < 36 in the spinal items of the ALSFRS-R at the time of PET at diagnosis. Overall, out of 665 potentially eligible subjects, 63 were included as PB and 271 as PS.

For a more comprehensive evaluation of patients’ brain metabolic changes, we included in the analyses 40 healthy controls (HC). We considered eligible as controls subjects referred to the PET centre for suspected lung cancer (i) with no oncologic disease detected, (ii) with brain PET scan reported as normal by the nuclear medicine physician, (iii) without history of neurological disorders, and (iv) with normal neurological examination.

### Genetic analysis

A repeat-primed PCR assay was used to screen for the presence of the GGGGCC hexanucleotide expansion in the first intron of *C9ORF72*. A cut-off of ≥ 30 repeats was considered pathological.

### 2-[^18^F]FDG-PET image acquisition and pre-processing

Brain 2-[^18^F]FDG-PET was performed according to published guidelines [12]. Patients fasted at least 6 h before the exam. Blood glucose was < 7.2 mmol/l in all cases before the procedure. After a 20-min rest, about 185 MBq of 2-[^18^F]FDG was injected. The acquisition started 60 min after the injection. PET/CT scans were performed on a Discovery ST-E System (General Electric). Brain CT and PET scan were sequentially acquired, the former being used for attenuation correction of PET data. The PET images were reconstructed with four iterations and 28 subsets with an initial voxel size of 2.34 × 2.34 × 2.00 mm, and data were collected in 128 × 128 matrices.

For ALS patients, a whole-body scan was performed setting head-first. Healthy controls underwent a separate brain scan after the whole-body one with a time difference of 15 min.

Images were spatially normalized to a customized brain 2-[^18^F]FDG-PET template [13] and subsequently smoothed with a 10-mm filter in MATLAB R2018b (MathWorks, Natick, MA, USA). Intensity normalization was performed at individual level averaging each voxel for the mean value of the whole brain.

### Statistical analysis

The demographic and clinical characteristics of patient groups (PB and PS ALS patients) and HC were compared as follows. The χ^2^-test was employed for categorical variables. The Mann–Whitney test was used for quantitative, continuous variables.

For the PET analyses, we compared PB and PS ALS patients employing the two-sample t test model of SPM12, including age at PET, sex, *C9ORF72* status, and King’s stage at PET as covariates. King’s stage was included as covariate since it influences brain metabolism [14]. We also compared each patient group with HC, including age at PET and sex as covariates. In all group comparisons, the height threshold was set at *P* < 0.001 (*P* < 0.05 FWE-corrected at cluster level). Only clusters containing > 125 contiguous voxels were considered significant. Brodmann areas (BAs) were identified at a 0–2-mm range from the Talairach coordinates of the SPM output isocentres corrected by Talairach Client (http://www.talairach.org/index.html).

Metabolic clusters showing a statistically significant difference between PB and PS patients underwent further analyses to test their accuracy in discriminating the two groups. To this end, all patients, regardless of the group they belonged to, underwent the following two-stage pre-processing: (i) metabolic levels have been normalized by the average of the whole brain region to avoid intrinsic noise in different measurements; (ii) from the normalized PET scan, the selected voxels have been retained (corresponding to the clusters obtained from the first-step analyses). This led to a dataset with 334 patients, where each patient was unambiguously identified by the *n* voxels of the selected clusters and the classification (PB/PS).

In order to address whether the above-selected clusters were indeed meaningful to discriminate PB and PS patients, a first experiment involved a full leave-one-out cross-validation (LOOCV) over the entire dataset. To broaden the investigation, four different classifiers were considered for comparison:

1. Support vector machines
2. K-nearest neighbours
3. Linear classifier
4. Decision tree

In order to automatize the hyperparameters tuning phase, each classifier underwent a Bayesian optimization stage. The optimizer was configured as follows:

- Maximum number of 3000 iterations;
- The objective function, to be maximized, reads as the LOOCV informedness J, defined as:
  1 $${\text{J }} = {\text{ Specificity }} + {\text{ Sensitivity }} - {1}$$

In order to further address the generalization capability of the model, we used a separate test set (i.e. hold-out set). The test set was composed of 10% of the patients, with the remaining 90% composing the training set, 33 patients being part of the test set and the remaining 301 being part of the training set. The training-and-test splitting procedure was performed randomly, yet with stratification. Indeed, the stratification ensured that both training and test set had the same proportion of pure bulbar and pure spinal subjects. The optimization procedure involved the training set only. Each of the four classifiers underwent a cross-validation on the training set in order to maximize Eq. (1). At the end of the optimization procedure, the model was trained on the full training set using the best hyperparameters and then validated on the test set.

In order to account for the randomness in the training-and-test set splitting, results are averaged across ten different training/test set random splits.

## Results

### Demographic, clinical and genetic data

The comparison of demographic and clinical data of PB, PS, and HC is reported in Table 1. We found significant differences between PB and PS in age at PET, sex, and, as expected, in ALSFRS-R total score and King’s stage at PET. However, the impact of these variables was kept under control including them as covariates in the analyses. In the comparisons with HC, the only significant difference was in male/female ratio between PB and HC. Also the effect of sex on the results was kept under control including it as covariate in the analyses with HC, as well as age at PET.

**﻿Table 1:** Comparison of demographic and clinical data of PB, PS, and HC

	PB	PS	HC	PB vs PS	PB vs HC	PS vs HC
	Median (IQR)	Median (IQR)	Median (IQR)	p*	p*	p*
Age at PET (years)	67.0 (60.0–72.0)	61.0 (53.0–69.0)	65.5 (55.0–72.0)	**0.001**	0.535	0.123
ALSFRS-R total score at PET	45.0 (44.0–46.0)	42.0 (39.0–45.0)		** < 0.001**		
	*n* (%)	*n* (%)	*n* (%)	*p*^§^	*p*^§^	*p*^§^
Sex						
Female	43 (68.3)	90 (33.2)	11 (27.5)	** < 0.001**	** < 0.001**	0.472
Male	20 (31.7)	181 (66.8)	29 (72.5)			
King’s stage						
Stage 1	63 (100.0)	168 (62.0)		** < 0.001**		
Stage 2	0 (0.0)	103 (38.0)				
Genetic status						
*C9ORF72* positive	8 (12.7)	23 (8.5)		0.299		
*C9ORF72* negative	55 (87.3)	248 (91.5)				
Total	63 (100.0)	271 (100.0)	40 (100.0)			

*PB,* pure bulbar patients; *PS,* pure spinal patients; *HC,* healthy controls. *Mann–Whitney *U* test. §Pearson’s Chi-squared test

### *2-[*^*18*^*F]FDG-PET data*

#### PB versus PS

**Fig. 1:**
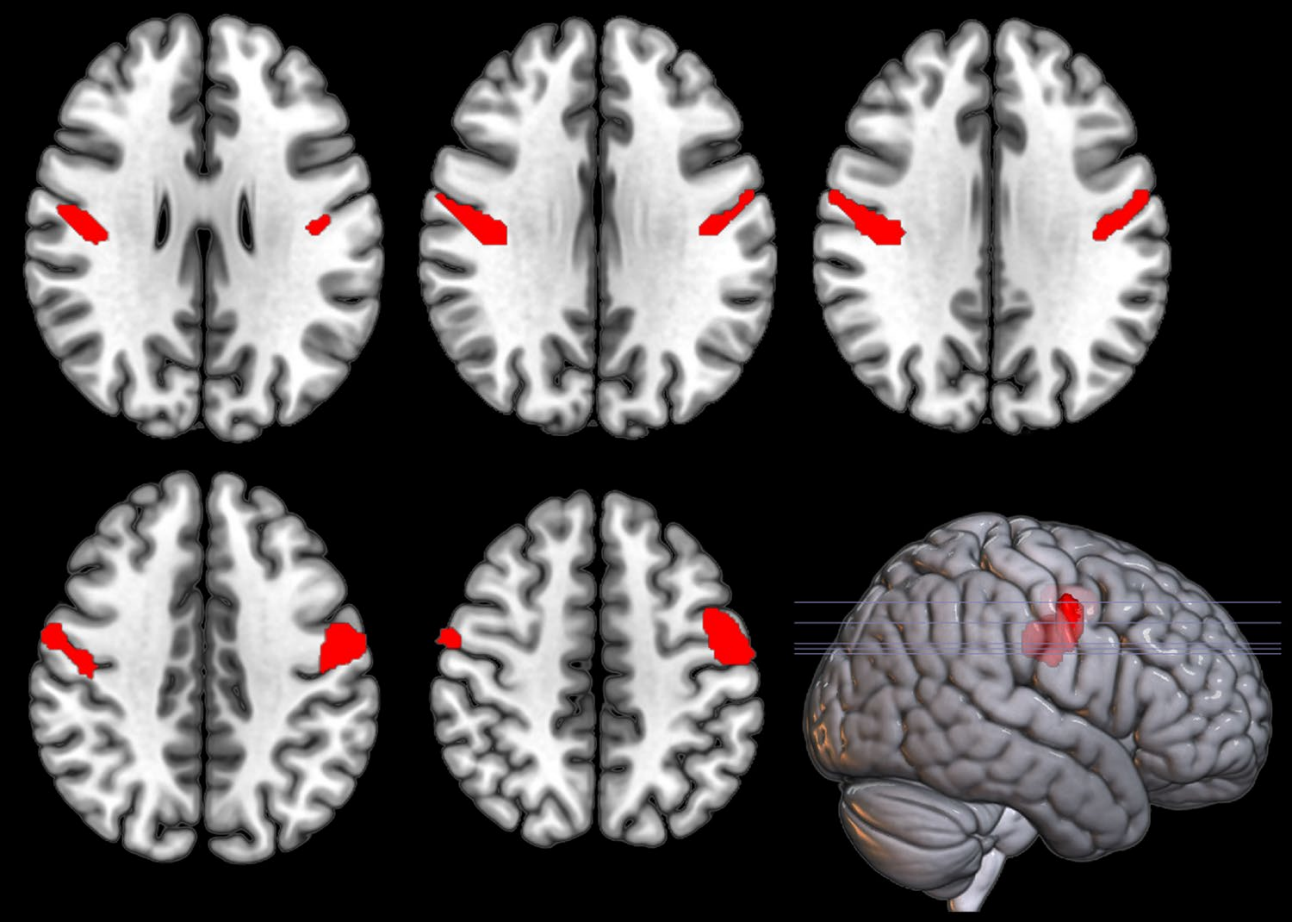
Pure Bulbar patients (PB) versus Pure Spinal (PS) patients. Clusters of relative hypometabolism of PB as compared to PS are marked in red on two-dimensional (2D) representation of transversal slices and on 3D rendering projections of the whole brain according to the MNI referential atlas (MRIcroGL software, https://www.nitrc.org/projects/mricrogl)

PB patients showed a relative hypometabolism compared to PS cases in bilateral precentral gyrus in correspondence with the regions of the motor cortex involved in the control of bulbar function (Table 2, Fig. 1). We did not find any significant cluster of relative hypometabolism in PS cases compared to PB patients.

**Table 2:** Clusters showing a statistically significant relative hypometabolism in PB patients compared to PS patients

P (FWE-corrected)	Cluster extent	*Z*-score	Talairach coordinates			Lobe	Cortical region	BA
0.009	763	5.94	− 53	− 6	43	Frontal	Left Precentral Gyrus	4
0.042	495	4.30	48	− 12	30	Frontal	Right Precentral Gyrus	6
		4.02	57	− 2	41	Frontal	Right Precentral Gyrus	6

*PB,* pure bulbar patients; *PS,* pure spinal patients; *BA,* Brodmann area

#### PB patients versus healthy controls

PB subjects showed a relative hypometabolism compared to HC in clusters including bilateral frontal and left temporal and occipital cortices (Supplemental Table 1, Supplemental Fig. 1). No cluster of relative hypermetabolism was found in PB compared to HC.

#### PS patients versus healthy controls

PS subjects showed a relative hypometabolism compared to HC in clusters including frontal and occipital cortices (Supplemental Table 2, Supplemental Fig. 2). No cluster of relative hypermetabolism was found in PS compared to HC.

### Leave-one-out cross-validation

In Table 3, we show the LOOCV error rate (expressed in percentage) at the end of the optimization stage (i.e. with the best hyperparameters for each classifier).

**Table 3:** Pure spinal versus pure bulbar—LOOCV error rate [%]

Classifier	Error rate
K-nearest neighbours	12.57
Decision tree	14.37
Support vector machine	4.19
Linear classifier	20.06

### Hold-out validation

In Table 4, we show the results in terms of error rate on the test set.

**Table 4:** Pure spinal versus pure bulbar—10% hold-out error rate [%]

Classifier	Error rate
K-nearest neighbours	10.91 ± 2.56
Decision tree	14.24 ± 4.3
Support vector machine	9.09 ± 2.02
Linear classifier	24.55 ± 20.12

## Discussion

The present study was focused on the evaluation of brain metabolic changes that differentiate ALS patients with PB and PS clinical involvement. The main finding was a relative hypometabolism in motor cortices in PB compared to PS patients, closely overlapping with the somatotopic representation of bulbar functions in the motor homunculus described by Penfield and Boldrey. Both PB and PS showed extensive hypometabolism as compared to HC, involving extramotor regions, in agreement with previous findings from our centre [6]. Our results are consistent with the hypothesis that ALS starts as a focal process [2] and its spread gives rise to the heterogeneity of motor phenotype. Some neuroimaging studies supported the concept of disease focality in vivo. An MRI study based on voxel-based morphometry showed that bulbar onset patients were characterized by bilateral focal atrophy in the bulbar segment of the homunculus of motor cortex, compared with patients with spinal onset, who displayed focal cortical changes in the limb segment of their motor strip [3]. Similarly, the site of onset (bulbar/spinal) resulted to be associated with the thinning of the corresponding part of the primary motor cortex in other MRI studies [4, 5]. Previous brain 2-[^18^F]FDG-PET works comparing bulbar and spinal onset patients showed divergent findings. An investigation from our centre including 32 patients identified large relatively hypometabolic clusters in bulbar onset patients as compared with spinal onset ones in prefrontal and frontal cortices [7]. A subsequent study based on the enlargement of the previous dataset (*n* = 195 ALS subjects) reported a relative hypometabolic cluster in the left motor and premotor cortex in bulbar onset patients as compared to spinal onset ones [6]. A work performed in a different population did not detect any difference between patients with bulbar and spinal onset [8]. In this context, a study aiming at testing the diagnostic accuracy of 2-[^18^F]FDG-PET in discriminating spinal and bulbar onset ALS patients showed poor performance [9]. Nevertheless, in this study a number of spinal onset patients had bulbar impairment and vice versa at the time of PET. Focusing on patients without functional evidence of the spread of the disease from spinal to bulbar regions and the other way round, in the present study, we demonstrated that the metabolic clusters including the regions representing bulbar function in the motor strip can discriminate between pure bulbar and pure spinal patients with an error rate < 10%. Namely, PB patients showed a relative hypometabolism as compared to PS ones in such regions. This finding seems in agreement with a study investigating disease spreading in ALS through the assessment of clinical evidence of upper and lower motor neuron signs [15]. The authors suggested that when the disease had a limb onset, bulbar neurons were more resistant to be involved as compared with other spinal regions. Moreover, neurophysiological data pointed towards a more relevant cortical dysfunction in bulbar onset ALS compared to limb onset disease, as expressed by hyperexcitability, possibly associated with loss of GABAergic inhibitory interneurons [16]. Notably, a recent MRI study employed VBM and tract-based spatial statistics (TBSS) to evaluate patterns of grey and white matter changes in bulbar and limb onset ALS. The authors suggested that bulbar onset ALS probably originates from the orofacial segments of the primary motor cortex, and that limb onset ALS patients have fewer cerebral structural integrity loss in the early phase of the disease, possibly due to a lower degree of neuronal network integration of the first pathologically affected regions [17]. The lack of clusters of relative hypometabolism in PS subjects compared to PB ones is challenging to interpret. Similarly to our results, a recent MRI study [5] found a significant cortical thinning of the bulbar regions of the motor strip in bulbar onset patients compared to spinal onset ones. Conversely, they did not detect any area of reduced thickness in the motor cortex of patients with spinal onset ALS compared to cases with bulbar onset. A possible explanation of these findings might be the prevalence of a dying-back mechanism in PS patients, leading to a relatively higher damage of lower compared to upper motor neurons. Despite some studies supporting this hypothesis [18], it remains speculative. Further data are necessary to clarify this issue and appropriately define the possible role of brain 2-[^18^F]FDG-PET as a biomarker in clinical trials. Taken together, our findings and literature data suggest that bulbar onset ALS is associated with an increased vulnerability of the bulbar regions of the motor cortex. The high discriminant value of the metabolism of the clusters identified in the present study provides some hints for further research. First, it supports the concept of the focal onset of the disease, whose phenotypic heterogeneity could be modulated by other factors. Second, it underlines that neuroimaging techniques can be implemented with other biomarkers to stratify patients in clinical trials towards a precision medicine approach. It is worth noting that the adoption of a high explainability (white box) artificial intelligence approach allowed to derive mechanistically relevant information from the model.

Our study has some limitations. First, to classify patients as PB or PS, we used the ALSFRS-R score, which is a functional scale and cannot disclose smooth clinical alterations detectable with a full neurological examination. Second, we lack a measure of upper motor neuron burden, allowing further analysis to highlight motor cortex dysfunction in PB patients. Third, our data are cross-sectional, while longitudinal data are necessary to place the focality of the onset in the context of the spread of the disease. Fourth, we did not perform partial volume effect correction for cortical atrophy, because we did not have MRI scans of the whole sample. However, previous studies employing voxel-based atrophy correction of resting glucose metabolism showed that metabolic measurements were relatively independent of brain atrophy [19].

In conclusion, by using cross-sectionally a single biomarker, we found clusters of relative hypometabolism in bilateral motor cortex in PB compared to PS patients, closely overlapping with the somatotopic representation of bulbar functions in the motor homunculus. The metabolism of such regions showed very high capability to discriminate between PB and PS patients. Our data provide in vivo support for the concept of the focality of ALS onset and strengthen the idea that 2-[^18^F]FDG-PET can play a role as a biomarker for precision medicine oriented clinical trials.

## Supplementary Information

Below is the link to the electronic supplementary material.

Supplementary file1 (PDF 136 KB)

Supplementary file2 (PDF 588 KB)

## Data Availability

The NIfTI files of the discriminant clusters will be available on demand by interested researchers.
