# Supplementary material for: Brain metabolic differences between pure bulbar and pure spinal ALS: a 2-[18F]FDG-PET study
Source: J Neurol. 2022 Nov 2;270(2):953–9. doi: 10.1007/s00415-022-11445-9 (PMC9886651; doi:10.1007/s00415-022-11445-9)

Journal name:

Journal of Neurology

Article Title:

Brain metabolic differences between pure bulbar and pure spinal ALS: a 2-[^{18}F]FDG-PET study

Authors:

Antonio Canosa, MD, PhD[1,2,3]; Alessio Martino, PhD[3,4]; Alessandro Giuliani, PhD[5]; Cristina Moglia, MD, PhD[1,2]; Rosario Vasta, MD[1]; Maurizio Grassano, MD[1]; Francesca Palumbo, MD[1]; Sara Cabras, MD[1]; Francesca Di Pede, MD[1]; Filippo De Mattei, MD[1]; Enrico Matteoni, MD[1]; Giulia Polverari, MD[6]; Umberto Manera, MD[1,2]; Andrea Calvo, MD, PhD[1,2,7]; Marco Pagani, MD, PhD[3,8]; Adriano Chiò, MD, FAAN[1,2,3,7]

Equal Author Contributions: Marco Pagani and Adriano Chiò contributed equally to this work.

Corresponding Author

Antonio Canosa, MD, PhD

ALS Centre, 'Rita Levi Montalcini' Department of Neuroscience, University of Turin

Via Cherasco 15, Turin, Italy, 10126

Phone +39(0)116335439

Fax +39(0)116336454

antonio.canosa@unito.it

ORCID ID: https://orcid.org/0000-0001-5876-4079

Affiliations

1. ALS Centre, ‘Rita Levi Montalcini’ Department of Neuroscience, University of Turin, Turin, Italy

2. Azienda Ospedaliero-Universitaria Città della Salute e della Scienza di Torino, SC Neurologia 1U, Turin, Italy

3. Institute of Cognitive Sciences and Technologies, C.N.R., Rome, Italy

4. Department of Business and Management, LUISS University, Rome, Italy

5. Environment and Health Department, Istituto Superiore di Sanità, Rome, Italy

6. Positron Emission Tomography Centre AFFIDEA-IRMET S.p.A., Turin, Italy

7. Neuroscience Institute of Turin (NIT), Turin, Italy

8. Department of Medical Radiation Physics and Nuclear Medicine, Karolinska University Hospital, Stockholm, Sweden

Supplemental Material

Supplemental Table 1. Clusters showing a statistically significant relative hypometabolism in PB compared to HC.

p(FWE-corrected)	Cluster extent	Z-score	Talairach coordinates			Lobe	Cortical region	BA
0.000	7803	6.22	-50	-2	42	Frontal	Left Precentral Gyrus	6
		5.17	-10	35	42	Frontal	Left Medial Frontal Gyrus	8
		4.99	-36	6	48	Frontal	Left Middle Frontal Gyrus	6
0.000	2119	4.90	-59	-5	-23	Temporal	Left Fusiform Gyrus	20
		4.25	-63	-6	-3	Temporal	Left Middle Temporal Gyrus	21
		4.05	-57	-14	-13	Temporal	Left Inferior Temporal Gyrus	21
0.000	1508	4.60	-36	-76	-1	Occipital	Left Inferior Occipital Gyrus	19
		3.46	-22	-91	5	Occipital	Left Middle Occipital Gyrus	18
		3.42	-10	-79	9	Occipital	Left Cuneus	17
0.019	603	4.41	14	52	29	Frontal	Right Superior Frontal Gyrus	9
		3.90	18	61	15	Frontal	Right Superior Frontal Gyrus	10
		3.83	26	34	52	Frontal	Right Superior Frontal Gyrus	8
0.000	2072	4.32	57	-4	37	Frontal	Right Precentral Gyrus	6
		4.27	46	-16	36	Frontal	Right Precentral Gyrus	4
		4.21	40	-1	53	Frontal	Right Middle Frontal Gyrus	6

PB: pure bulbar patients. HC: healthy controls. BA: Brodmann Area.

Supplemental Table 2. Clusters showing a statistically significant relative hypometabolism in PS patients compared to HC.

P (FWE-corrected)	Cluster extent	Z-score	Talairach coordinates			Lobe	Cortical Region	BA
0.000	14903	6.54	-20	-93	3	Occipital	Left Cuneus	17
		6.44	22	-93	8	Occipital	Right Middle Occipital Gyrus	18
		6.24	8	-91	5	Occipital	Right Cuneus	17
0.000	7625	5.29	-20	39	-26	Frontal	Left Orbital Gyrus	11
		5.23	20	35	-30	Frontal	Right Orbital Gyrus	11
		5.14	-40	26	23	Frontal	Left Middle Frontal Gyrus	46
0.001	1126	4.51	44	6	40	Frontal	Right Middle Frontal Gyrus	6
		3.66	46	30	22	Frontal	Right Middle Frontal Gyrus	46
		3.61	53	36	11	Frontal	Right Inferior Frontal Gyrus	46
0.009	752	3.93	34	48	-2	Frontal	Right Middle Frontal Gyrus	10
		3.89	14	59	14	Frontal	Right Superior Frontal Gyrus	10

PS: pure spinal patients. HC: healthy controls. BA: Brodmann Area.

Supplemental Fig. 1

Pure Bulbar (PB) patients versus healthy controls (HC)

Clusters of relative hypometabolism of PB as compared to HC are marked in green on two-dimensional (2D) representation of transversal slices and on 3D rendering projections of the whole brain according to the MNI referential atlas (MRIcroGL software, https://www.nitrc.org/projects/mricrogl)

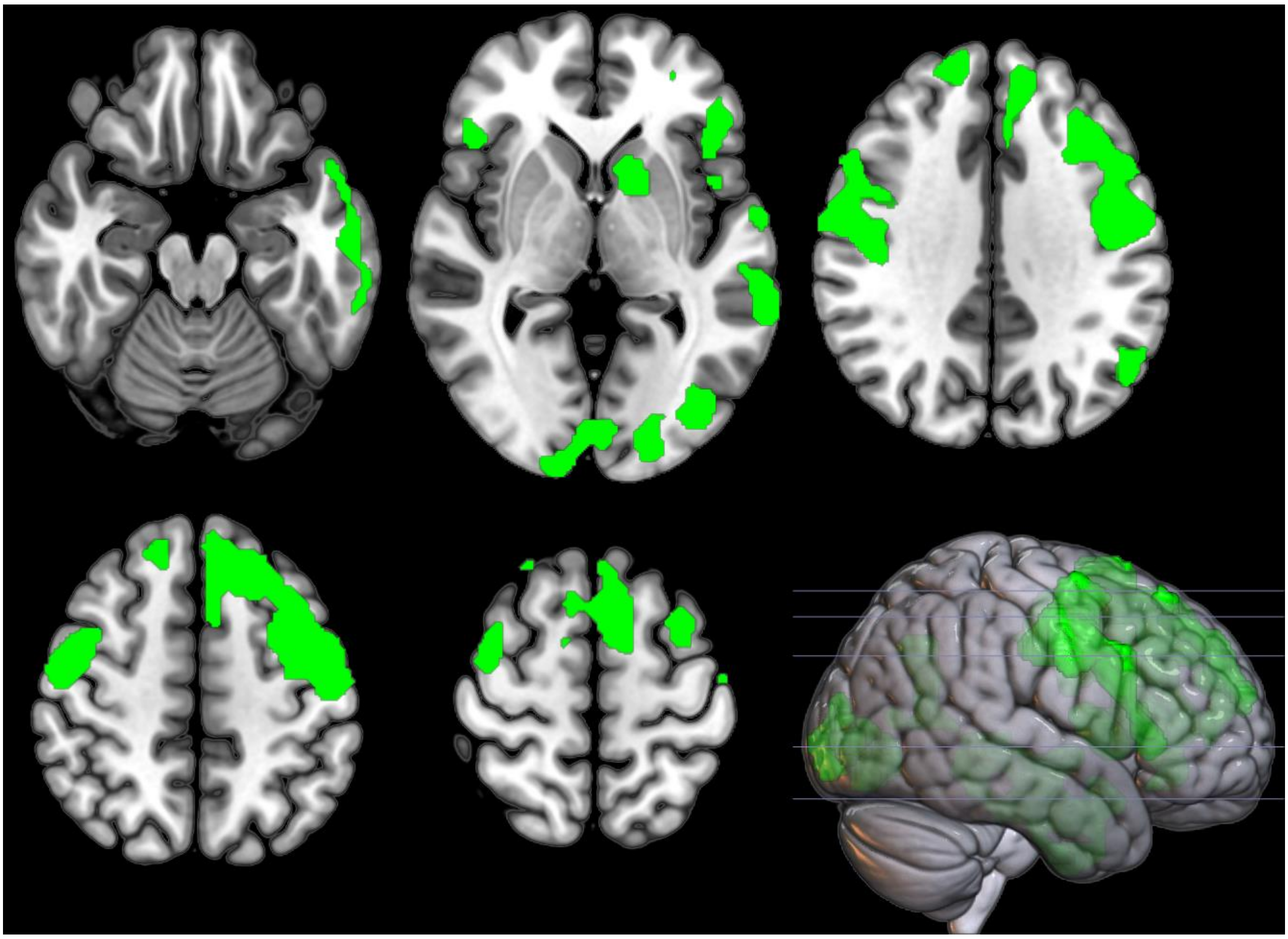

Supplemental Fig. 2

Pure Spinal (PS) patients versus healthy controls (HC)

Clusters of relative hypometabolism of PS as compared to HC are marked in blue on two-dimensional (2D) representation of transversal slices and on 3D rendering projections of the whole brain according to the MNI referential atlas (MRIcroGL software, https://www.nitrc.org/projects/mricrogl)

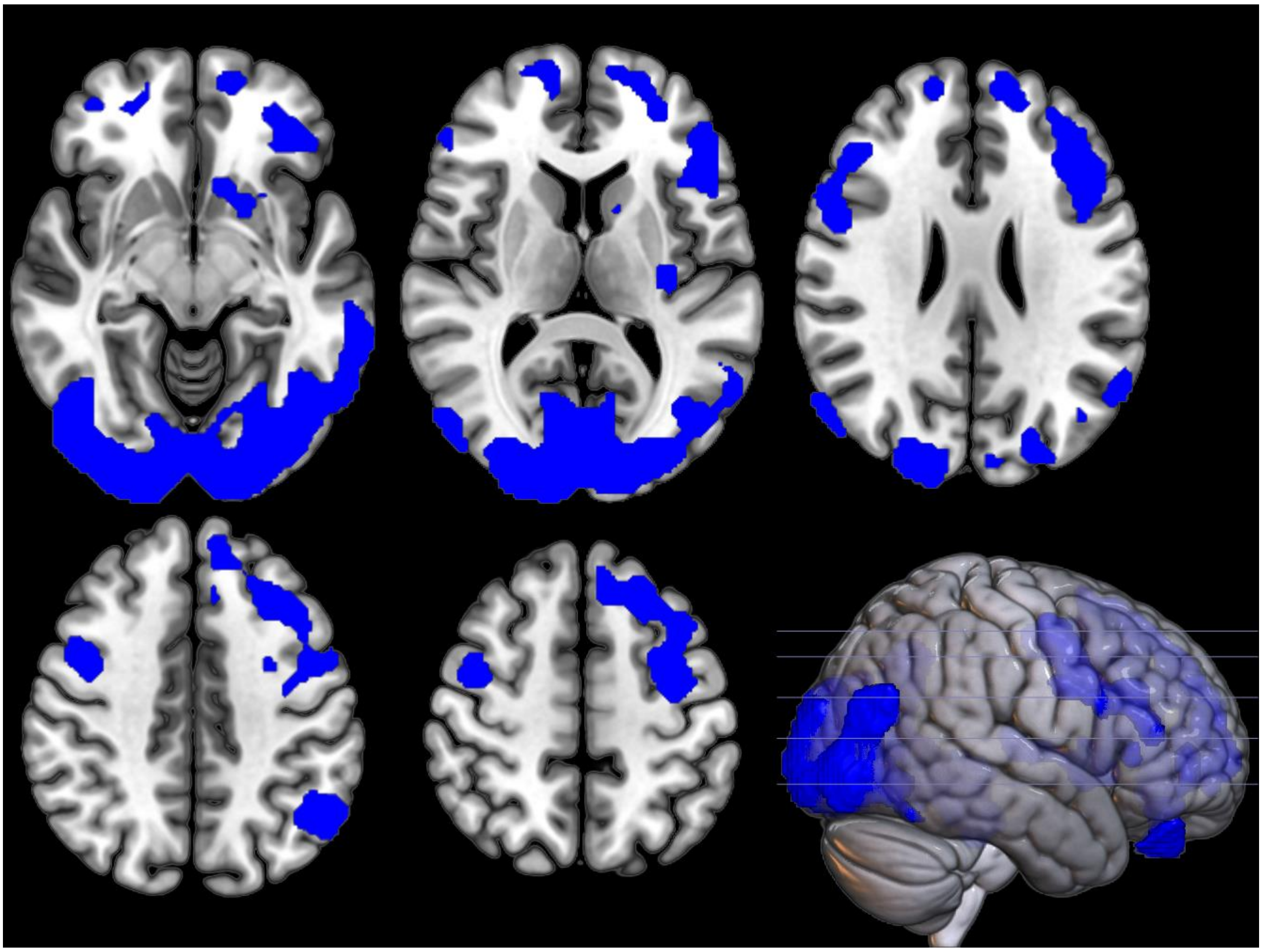

Supplement: Supplementary file 2 — Supplementary file2 (PDF 588 KB) [file 415_2022_11445_MOESM2_ESM.pdf]
